# Distribution pattern of amino acid mutations in chloroquine and antifolate drug resistance associated genes in complicated and uncomplicated *Plasmodium vivax* isolates from Chandigarh, North India

**DOI:** 10.1186/s12879-020-05397-6

**Published:** 2020-09-15

**Authors:** Hargobinder Kaur, Rakesh Sehgal, Archit Kumar, Praveen K. Bharti, Devendra Bansal, Pradyumna K. Mohapatra, Jagadish Mahanta, Ali A. Sultan

**Affiliations:** 1 Department of Medical Parasitology, Postgraduate Institute of Medical Education and Research, Chandigarh, India; 2 Department of Virology, Postgraduate Institute of Medical Education and Research, Chandigarh, India; 3 National Institute for Research in Tribal Health, Indian Council of Medical Research, Nagpur Road, Garha, Jabalpur, Madhya Pradesh India; 4 Department of Microbiology and Immunology, Weill Cornell Medicine – Qatar, Cornell University, Qatar Foundation, Education City, Doha, Qatar; 5 Present address: Ministry of Public Health, Doha, Qatar; 6 Regional Medical Research Centre, NE, Indian Council of Medical Research, Post Box no.105, Dibrugarh, Assam India

**Keywords:** *Plasmodium vivax*, Complicated malaria, Antimalarial drug resistance, North India

## Abstract

**Background:**

The increasing antimalarial drug resistance is a significant hindrance to malaria control and elimination programs. For the last six decades, chloroquine (CQ) plus pyrimethamine remains the first-line treatment for *P. vivax* malaria. Regions where both *P. falciparum* and *P. vivax* co-exist, *P. vivax* is exposed to antifolate drugs due to either misdiagnosis or improper treatment that causes selective drug pressure to evolve. Therefore, the present study aims to estimate antimalarial drug resistance among the complicated and uncomplicated *P. vivax* patients.

**Methods:**

A total of 143 *P. vivax* malaria positive patients were enrolled in this study, and DNA was isolated from their blood samples. Pvcrt-o, Pvmdr-1, Pvdhps, and Pvdhfr genes were PCRs amplified, and drug resistance-associated gene mutations were analyzed. Statistical analysis of the drug resistance genes and population diversity was performed using MEGA vs. 7.0.21 and DnaSP v software.

**Results:**

Among the CQ resistance marker gene Pvcrt-o, the prevalence of K10 insertion was 17.5% (7/40) and 9.5% (7/73) of complicated and uncomplicated P vivax group isolates respectively. In Pvmdr-1, double mutant haplotype (**M**_**958**_**/L**_**1076**_) was found in 99% of the clinical isolates. Among the pyrimethamine resistance-associated gene Pvdhfr, the double mutant haplotype I_13_P_33_F_57_**R**_**58**_T_61_**N**_**117**_I_173_ was detected in 23% (11/48) in complicated and 20% (17/85) in uncomplicated group isolates. In the sulphadoxine resistance-associated Pvdhps gene, limited polymorphism was observed with the presence of a single mutant (D459A) among 16 and 5% of the clinical isolates in the complicated and uncomplicated group respectively.

**Conclusion:**

The study presents the situations of polymorphism in the antimalarial drug resistance-associated genes and emphasizes the need for regular surveillance. It is imperative for the development of suitable antimalarial drug policy in India.

## Background

Malaria is a world’s most widespread febrile illness and causes significant public health concern in morbidity and mortality. Approximately 85% of the global infectious disease burden has been attributed to malaria and six other infectious diseases. As per the recent WHO estimates, 219 million cases reported in 91 countries with an estimated mortality of 405,000 [1]. Out of the total *P. vivax* malaria cases reported in 2019, 53% of *P. vivax* burden is in the WHO South-East Asia Region, and around 47% of them in India alone [1]. Although severe malaria is known to be associated with *P. falciparum*, in the last decade, reports have highlighted severe *P. vivax* malaria, which was earlier considered benign [2, 3]. Several studies have reported severe life-threatening symptoms in *P. vivax* patients from Asia, South America, and Africa [4, 5]. In India, a more extensive series of studies have associated 9–68% of severe *P. vivax* infections with a severe and fatal disease in both children and adults [5–10]. The complications related to severe *P. vivax* malaria include the symptoms of altered sensorium, seizures, cerebral malaria, jaundice, acute respiratory distress syndrome (ARDS), shock, acute kidney injury (AKI), and severe anemia [5, 9]. Mechanisms underlying the biology, pathogenesis and epidemiology of the severe vivax syndromes remain poorly understood and require further investigation.

The clinical outcome of malaria is thought to be mainly contributed by the host, parasite and environmental factors [11]. India aims to eliminate malaria nationally but has struggled to do so. Despite all the measures for the control and elimination of malaria, the biggest hurdle in the path is the increasing resistance to insecticides and antimalarials to the growing trend of population migration [12]. In India, first-line treatment for the uncomplicated *P. vivax* malaria includes the combination of chloroquine (CQ, for eliminating blood stages) and primaquine for liver stages (hypnozoites). Currently, complicated *P. vivax* malaria cases are subjected to Artemisinin combination therapy [13]. These combinations of drugs remain overall effective, but with the reports of resistance from the past few years makes malaria control and elimination a difficult task [14, 15]. Regardless of several clinical reports, the true estimates of the antimalarial resistance have been poorly defined. A few therapeutic assessment studies from southwestern and eastern India have reported the outstanding efficacy of CQ in treating uncomplicated *P. vivax* malaria patients with 0.8% (1/125) of therapeutic failure [16–18]. Unlike *P. falciparum*, in *P. vivax* in view of the various confounding factors like the variant immune status, reinfection, frequent relapses and the lack of continuous in-vitro culture methods, the therapeutic efficacy studies and in-vitro susceptibility assays is always cumbersome to conduct [19]. In order to monitor the drug resistance in *P. vivax*, the molecular markers in the malaria parasites are considered as one of the important tools [20]. In *P. vivax*, Pvmdr1 and Pvcrt have been recognized as homolog of Pfmdr1 and Pfcrt and it has been linked in the modulation of chloroquine susceptibility [19, 21]. In the areas of co-endemicity, Sulfadoxine/pyrimethamine (SP) had been rigorously used for the treatment of *P. falciparum* and severe malaria, enabling the selection of resistant *P. vivax* [22]. An increased morbidity rate has been reported due to *P. vivax* malaria in southeast Asia, with the emergence and spread of less susceptible strains of *P. vivax* to antifolate drugs [23]. Mutations in the *P. vivax* ortholog enzymes Pvdhfr and Pvdhps targeted by SP have also been identified in the areas of *P. falciparum* treated with combinational therapy consisting of SP and are linked to decreased sensitivity to Sulphadoxine-pyrimethamine (SP) [22]. The ever-expanding burden of severe *P. vivax* infections together with the drug resistance could result in an enormous expansion of the fatal infection similar to *P. falciparum* [24]. Hence, it becomes essential to study the current status of the chloroquine and SP drug resistance in *P. vivax* cases. In the present study, we assessed the CQ (Pvcrt-o and Pvmdr-1) and SP (Pvdhfr and Pvdhps) drug resistance patterns among the complicated and uncomplicated *P. vivax* isolates. The baseline molecular information on CQ and antifolate drug resistance will help in formulating the future drug policy for malaria in India.

## Methods

### Study subjects and sample collection

The present study was carried out at the Postgraduate Institute of Medical Education and Research (PGIMER, Chandigarh) from a period of 2013 to 2016, and a total of 143 *P. vivax* positive samples were recruited. Intravenous blood sample were collected in a sterile EDTA vacutainer by a trained practitioner. All the malaria suspected patients who had fever > 37.5 °C or a history of fever within the previous 24–48 h were further tested for *P. vivax* by microscopy/ antigen detection/ nested PCR before enrollment into the study. The samples were confirmed for *P. vivax* by molecular methods as described earlier by Kaur et al. [9]. The samples were classified as complicated and uncomplicated *P. vivax* on the basis of the WHO based criteria for severe malaria patients (viz., Renal impairment: Plasma or serum creatinine≥3 mg/dl, Impaired Consciousness and Multiple convulsions and Jaundice: Plasma or serum Bilirubin ≥3 mg/dl) [25].

### DNA isolation and amplification of *Plasmodium vivax* drug resistance genes

The DNA from the whole blood samples of the patients were isolated using the QIAamp DNA blood mini kit as per manufacturer’s instructions (Qiagen, Valencia, CA, USA and stored at − 20 °C for further analysis. For the designing of primers for chloroquine resistance transporter (crt-o), dihydrofolate reductase-thymidylate synthase (DHFR-TS) and dihydropterin pyrophosphokinase-dihydropteroate synthase (PPPK-DHPS) genes, the reference sequence of *P. vivax* EU333972.1, EU478871.1, EU478858.1 were used [26]*.* The *P. vivax* multidrug resistance gene (*Pvmdr-1*) was targeted by using already published primer [27]. The sequences of the primers, and the respective product size of each gene are summarized in Additional file 1.

The PCRs amplification of the targeted genes (Pvcrt-o, Pvmdr-1, Pvdhps and Pvdhfr*)* were carried out for all *P. vivax* PCR positive patient samples. The negative control (nuclease free water) was included in each amplification reaction and precautions were taken to prevent cross-contamination. All the PCR reaction mixtures were prepared using high fidelity Platinum Taq DNA polymerase (Thermo Fisher Scientific, Inc., Wilmington, DE) as detailed in Additional file 2 and the thermocycler conditions used are summarized in the Additional file 3. PCR products were visualized on agarose gel stained with ethidium bromide under UV light.

### Nucleotide sequencing

The PCR products were purified using the Qiagen PCR purification kit as per the manufacturer’s instructions (QIAGEN, CA, USA). The purified products of all the four genes were sequenced bidirectionally using Sanger method (Genewiz INC, NJ, USA). Sequences were manually cured and Expasy translate software was used for translation of the sequences. Clustal X 2.1 was used for performing multiple sequence alignment (MSA) to see intraspecific variation (SNPs) if any, among the sequences on comparison with Sal-I reference sequence.

### Test of neutrality, selection pressure and statistical analysis

Two statistical tests (Tajima’s D-test (Tajima, 1989), D* and F* statistics of Fu’s and Li’s tests (Fu and Li, 1993)) were applied to test the hypothesis which states that the allele frequency range is compatible with the neutral model. MEGA vs 7.0.21 [28] and DnaSP ver 5.10.01 [29] softwares were used for performing statistical analysis for drug resistance genes (Pvcrt-o, Pvmdr-1, Pvdhps, Pvdhfr) in order to investigate the population diversity. The various genetic parameters determined were; π- Nucleotide diversity (per site), nucleotide diversity parameter, H- no. of haplotypes and Hd- haplotype diversity.

## Results

### Demographic and clinical details of the enrolled *P. vivax* patients

A total of 143 *P. vivax* malaria positive patients were enrolled and the clinical histories for all of them were collected at the time of sample collection. Five *P. vivax* patients were excluded from the study due to the presence of one or the other co-infection of scrub typhus, *Burkholderia cepacia* sepsis, Dengue, typhoid and one patient had acute lymphoblastic leukemia (ALL). All the patients with malaria were treated as per the National treatment guidelines [13]. The 143 *P. vivax* patients were categorized into two groups, complicated and uncomplicated, on the basis of WHO criteria for severe malaria. The majority of patients were found to have uncomplicated *P. vivax* malaria (64.3%; 92/143), while 35.7% (51/143) patients had complicated *P. vivax* malaria. The median age of the complicated and uncomplicated *P. vivax* patients was found to be 17.0 + SD (8–27) and 9.5+ SD (4–20) years with the male to female ratio of 1:0.6 and 1:0.5. The uncomplicated *P. vivax* malaria patients were presented with the symptoms of fever accompanied with chills and rigors, headaches, nausea, vomiting and general body weakness. Major complications present in the complicated group of patients were severe thrombocytopenia (23.5%; 12/51), hypotensive shock or hypovolemic shock (19.6%; 10/51), jaundice (17.6%; 9/51) followed by the altered sensorium with the involvement of CNS (13.7%; 7/51), multiple convulsions (13.7%; 7/51) and renal impairment in 11.7% (6/51).

### Mutation analysis of CQ and antifolate drug resistance associated genes

The nested and conventional PCRs were performed for Pvcrt-o, Pvmdr-1, Pvdhfr and Pvdhps genes for a total of 143 *P. vivax* positive samples. Pvcrt-o, Pvmdr-1, Pvdhfr and Pvdhps gene sequencing was successful in a total of 79% (113/143), 82.5% (118/143), 93% (133/143) and 86.7% (124/143) of *P. vivax* clinical isolates respectively. The multiple sequence alignment of the deduced protein sequence was performed for all the genes.

### Pvcrt-o and Pvmdr-1 mutation analysis

**Fig. 1:**
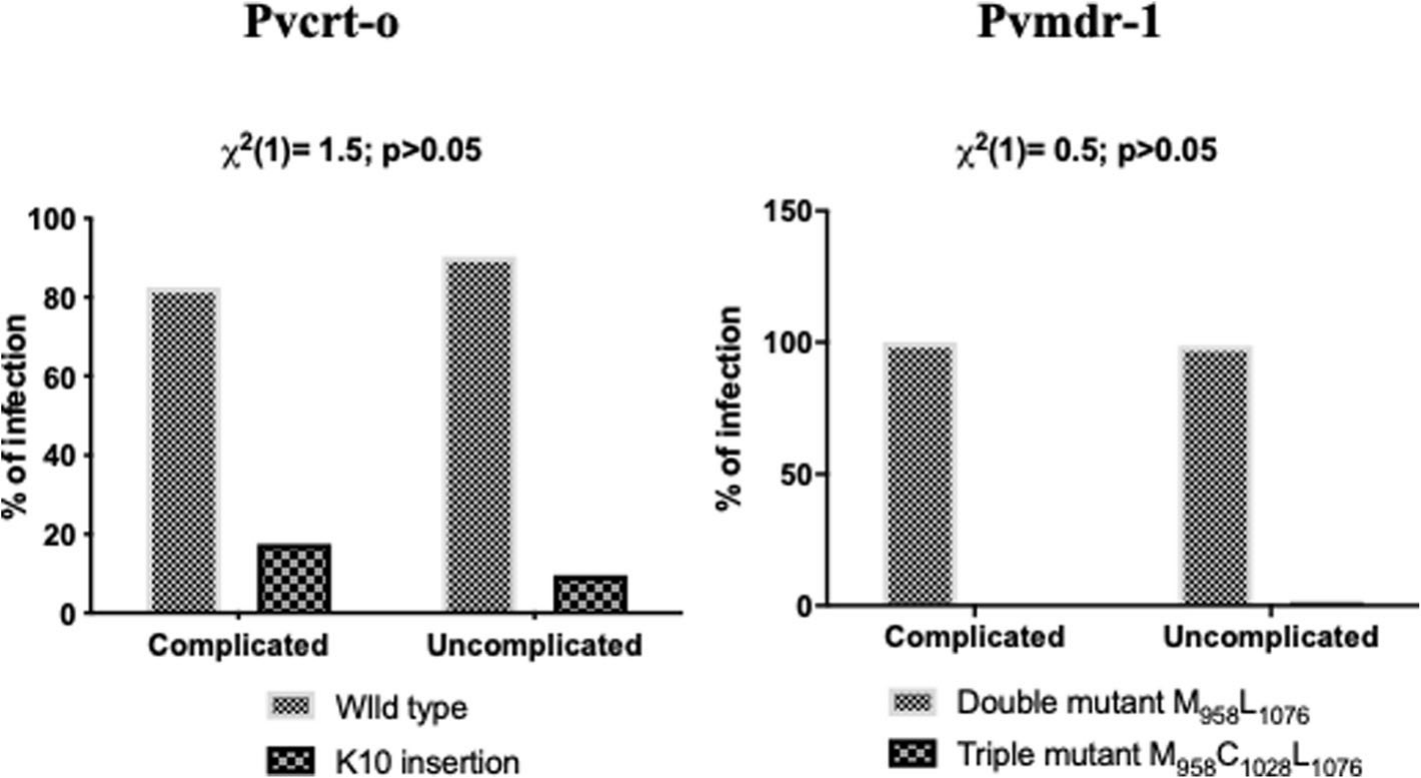
Mutation rates in *P. vivax* genes (Pvcrt-o *and* Pvmdr-1) conferring resistance to CQ

The obtained 1 kb sequence of Pvcrt-o and ~ 0.6 kb sequence of Pvmdr-1 gene for both complicated and uncomplicated group isolates were then compared with the Pvcrt-o and Pvmdr-1 reference Sal I sequence (GenBank: AF314649 and AY571984.1). A total of 17.5% (7/40) complicated cases had lysine (AAG) insertion at the 10th amino acid position of exon1 of Pvcrt-1 as compared to the uncomplicated cases, where only 9.5% (7/73) of the isolates had the K10 insertion. Sequence analysis of *Pvmdr-1* gene revealed the presence of double mutants (T958**M**/F1076**L)** and triple mutants (T958**M/** F1076**L/**Y1028**C)** in *P. vivax* isolates. Double mutations (T958**M** /F1076**L**) in *Pvmdr-1* gene was observed in 100% (41/41) of the complicated and 98.7% (76/77) of the uncomplicated isolates. A single triple mutant (T958**M**/F1076**L**/Y1028**C**) was observed only in uncomplicated *P. vivax* group isolate (Table 1). No statistically significant relatedness was (χ2 (1) = 1.5; *p* > 0.05) found with the presence of the K10 insertion in Pvcrt-o and double mutant (**M**_**958**_**L**_**1076**_) in Pvmdr-1 gene (χ2 (1) = 0.5; *p* > 0.05) with complicated *P. vivax* group (Fig. 1).

**Table 1:** Pvmdr-1 gene mutation analysis in complicated and uncomplicated group of *P. vivax* isolates

Pvmdr1	Complicated (***n*** = 41/51)	Uncomplicated (***n*** = 77/92)	Total (***n*** = 118/143)
**Wild type** (T_958_ Y_976_ Y_1028_ F_1076_)	0	0	0
**Double mutant** **M**_**958**_**L**_**1076**_ n (%)	41 (100)	76 (98.7)	117 (99.2)
**Triple mutant** M_958_C_1028_L_1076_ n (%)	0	1 (1.3)	1

### Pvdhps *and* Pvdhfr mutation analysis

**Fig. 2:**
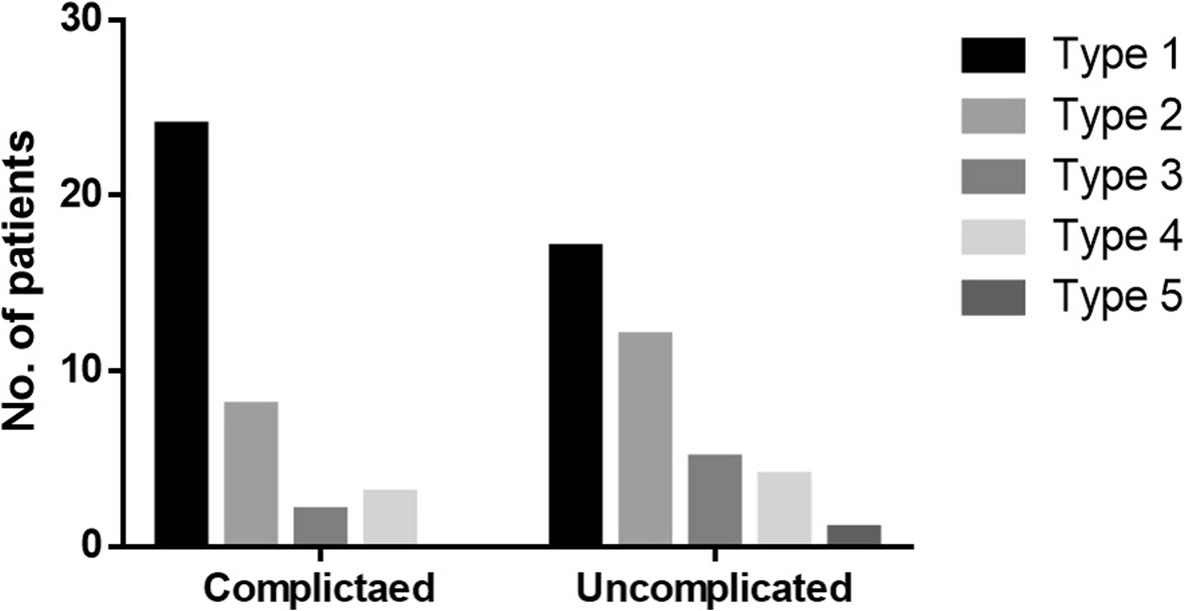
Distribution of GGDN repeats in Pvdhfr among complicated and uncomplicated group of patients

**Fig. 3:**
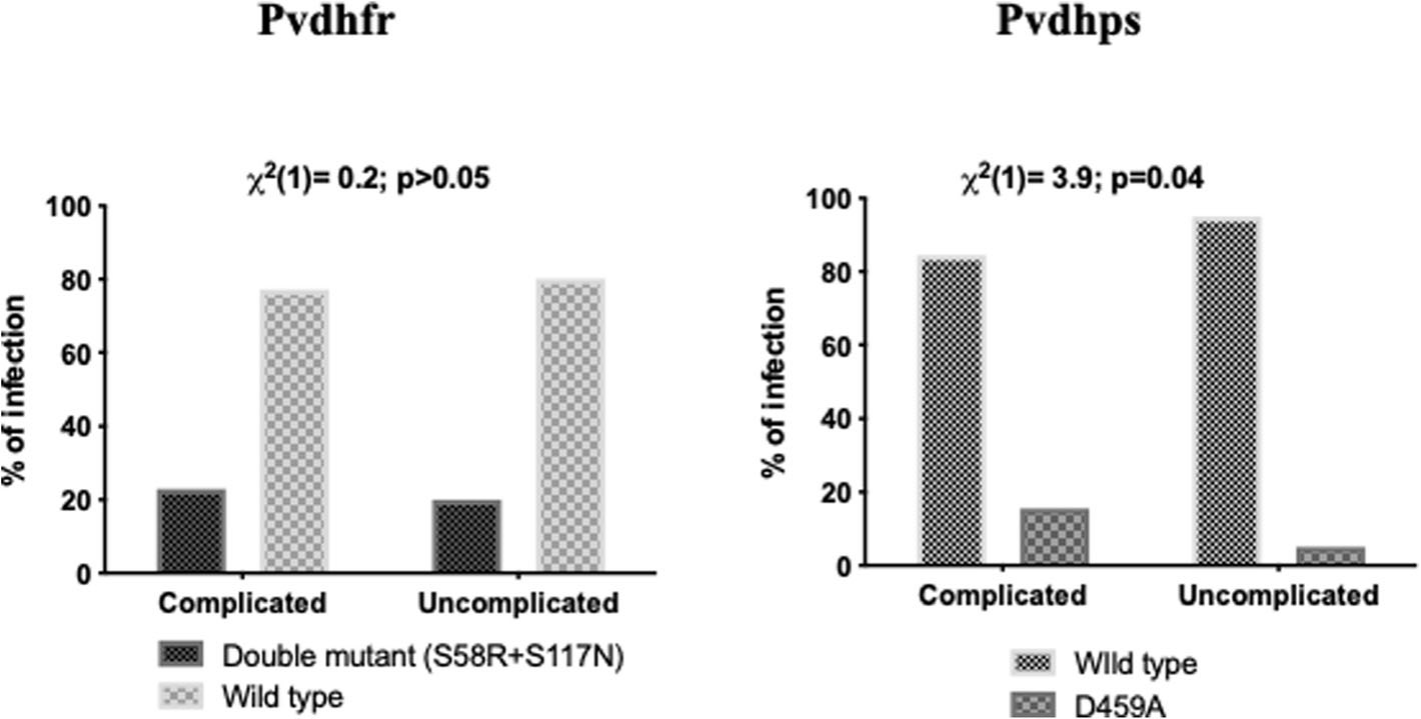
Mutation rates in *P. vivax* genes (Pvdhps and Pvdhfr) conferring resistance to SP drugs

Sequence analysis of Pvdhfr revealed the presence of double mutants (S58**R**/S117**N**) among the clinical isolates while other mutations F57L, T61S and I173L/F were found to be absent in comparison with the reference Sal I strain sequence (GenBank: X98123). The majority (78.9%; 105/133) of the isolates were of wild type at positions I_13_P_33_F_57_S_58_T_61_S_117_I_173_. However, among the complicated and uncomplicated group, double mutants **(**I_13_P_33_F_57_**R**_**58**_T_61_**N**_**117**_I_173_) was observed in 22.9% (11/48) and 20% (17/85) of the isolates, respectively (Table 2). None of the isolate was observed to consist of any triple or quadruple mutation. Of the sequenced isolates for Pvdhps, majority 84.4%; (38/45) and 94.9%; (75/79) of the isolates in both the complicated and uncomplicated groups were wild type. The non-synonymous mutation (D459A) was found to be present in 15.5% (7/45) and 5% (4/79) of the isolates from complicated and uncomplicated group and shows the absence of other SNPs (C422R and A553G). The Tandem repeat variants (TRV) observed in Pvdhfr were further classified into five types (Type 1, Type 2, Type 3, Type 4 and Type 5) based on the insertion/deletion of the GGDN at amino acid position 88 of Pvdhfr among the *P. vivax* isolates (Table 3). The Type 1 was found to be the most prevalent TRV among complicated (50%; 24/48) and uncomplicated (20%; 17/85) group, followed by the Type 2 among complicated (16.6%; 8/48) and uncomplicated (14.1%; 12/85) isolates as shown in Fig. 2. All the observed double mutants of Pvdhfr gene in both the groups carried the Type 1 TRV, whereas the other TRV types were observed to be present with the wild type alleles. Statistically significant relatedness was (χ2 (1) = 3.9; *p* = 0.04) found between the presence of the D459A in Pvdhps gene and the complicated *P. vivax* group, whereas no association was observed for Pvdhfr double mutants and complicated group (Fig. 3).

**Table 2:** Pvdhfr gene mutation analysis in complicated and uncomplicated group of *P. vivax* isolates

Pvdhfr	Complicated (***n*** = 48/51)	Uncomplicated (***n*** = 85/92)	Total (***n*** = 133/143)
Wild type (I_13_P_33_F_57_S_58_T_61_S_117_I_173_) N (%)	37 (77.1)	68 (80)	105 (78.9)
Double mutant I_13_P_33_F_57_**R**_**58**_T_61_**N**_**117**_I_173_ N (%)	11 (22.9)	17 (20)	28 (21.1)

**Table 3:** Tandem repeat variant (TRV) types of Pvdhfr

Types	GGDN repeats
Type 1	GGDNTS GGDNTH GGDNAD/N
Type 2	GGDNTH GGDNAD
Type 3	GGDNTS GGDNAD
Type 4	GGDNTS GGDNTH GGDNTH GGDNAD
Type 5	GGDNTH GGDNTH GGDNTH GGDNAD

### Genetic structure of antimalarial drug resistance associated markers

The analysis was extended to test Tajima’s D test of neutrality and Fu and Li′s D and F test statistics for Pvmdr-1*,* Pvdhps and Pvdhfr (Table 4). No role of the evolutionary natural selection was found in the Pvdhps and Pvdhfr drug resistance genes whereas the negative Fu and Li′s D and F test statistic value of Pvmdr-1 gene suggest the presence of evolutionary forces leading to the generation of new variants.

**Table 4:** Analysis of the test of neutrality (Tajima’s D and Fu and Li′s D and F test statistic) at Pvmdr-1, Pvdhr, Pvdhps

	Occurrence of mutation	H	Hd (±SD)	Nucleotide Diversity	Tajima’s D	Fu and Li′s D and F test statistic
Pvmdr-1	n (%)			π (±SD)		
Complicated	41 (100)	2	0.048 ± 0.045	0.00009 ± 0.00009	−1.11966	−1.78899; − 1.84602
Uncomplicated	77 (100)	4	0.081 ± 0.044	0.00015 ± 0.00008	−1.65144	−3.24282*; −3.21499*
Combined	118 (100)	3	0.034 ± 0.023	0.00007 ± 0.00005	−1.35708	−2.88867*; −2.82660*
**Pvdhfr**						
Complicated	11 (22.9)	6	0.713 ± 0.035	0.00228 ± 0.00024	−0.18017	−0.30060; −0.30785
Uncomplicated	17 (20)	3	0.614 ± 0.033	0.00165 ± 0.00016	1.38695	0.84089; 1.18451
Combined	28 (21)	5	0.652 ± 0.019	0.00182 ± 0.00012	0.51341	−1.18432; −0.73304
**Pvdhps**						
Complicated	7 (15.5)	3	0.341 ± 0.081	0.00064 ± 0.00016	−0.40771	0.75830;0.48349
Uncomplicated	4 (5)	3	0.164 ± 0.054	0.00031 ± 0.00010	−0.94808	−1.01710; −1.16199
Combined	11 (9)	3	0.229 ± 0.047	0.00044 ± 0.00016	−0.99655	−0.63719; − 0.63719

S = Number of polymorphic (segregating) sites

π = observed average pair-wise nucleotide diversity

Rm = Minimum number of recombination events

H = number of nucleotide haplotypes

Hd = haplotype diversity

* = *p* < 0.05

## Discussion

Various studies have reported the increase in CQ resistant *P. vivax* in New Guinea and Indonesia with declining reports of CQ efficiency from almost all the endemic countries [30]. The present situation of the increasing burden of severe *P. vivax* infections, delay in diagnosis, partially effective treatment regimens, and the arising antimalarial drug resistance could result in an enormous expansion of the fatal infection similar to *P. falciparum* [24]. The patterns of drug resistance of Pvcrt-o and Pvmdr-1 have been identified in the clinical isolates. A total of 17.5% (7/40) complicated vivax isolates was found to have lysine (AAG) insertion at the 10th amino acid position of exon1 of Pvcrt-1 as compared to the isolates of uncomplicated group 9.5% (7/73). The findings of the present study are in complete concordance with the recent study from India, which has reported (first time in India) the insertion of lysine residue (AAG) in the Pvcrt gene in 5.6% of the isolates [31]. In contrast to Indian studies, a very high prevalence of K10 insertion in the Pvcrt gene have been reported from Thailand (56–89%) and Myanmar (46–72%) [27, 31–33].

Pvmdr-1 gene analysis revealed the presence of double mutants (T958**M** /F1076**L**) in 100% (41/41) of the complicated and in 98.7% (76/77) of the uncomplicated group isolates, with the presence of a single triple mutant (T958**M**/F1076**L**/Y1028**C**) observed in isolates of *P. vivax* uncomplicated group of patients. The observed nucleotide diversity in Pvmdr-1 gene was present at low level (π = 0.00007 ± 0.00005) as compared to a previous study by Cubides et al. (π = 0.0013) [34]. The overall increased (12.4%) prevalence of AAG insertion in Pvcrt with the complete absence of Y976F in Pvmdr-1 was observed. Suwanarusk et al. reported a correlation between an increased CQ IC50 with the K10 insertion and Y976F mutation in Pvcrt-o and Pvmdr-1 gene [19]. In Pvmdr-1 gene, the overall dominance of double haplotype (**M**_**958**_**/L**_**1076**_) was seen in 99% of the clinical isolates in concordance with the earlier studies [31]. Khattak et al. have reported the complete absence of Y976F mutation in the Pvmdr-1 gene, with 98% of the isolates harboring F1076**L** mutation [28]. Previous studies have reported T958**M** mutation, localized in the transmembrane domain of the Pvmdr-1 gene, from countries having low to a high level of Chloroquine Resistance (CQR). Another mutation observed in our patient cohort was Y1028C in the Pvmdr-1 gene, which was observed in only 1.3% (1/77) of clinical isolate in the uncomplicated group of the patients. A recent Indian study by Joy et al. have reported a similar mutational frequency for Y1028C (1.2%) [31]. The present study results are consistent with the previous study’s results, showing a rise in the Pvmdr-1 F1076L prior to Y976F in the clinical isolates [35, 36]. If the hypothesis by Brega et al. of the two-step trajectory of mutations at codon F1076L followed by Y976F may be responsible for leading CQ resistance is true, then the F1076L observed in the study is a warning sign of emerging CQR in North India prior to the appearance of drug resistance phenotype in the population [36]. Studies have reported an association between the increased morbidity rate due to *P. vivax* with the emergence and spread of less susceptible strains of *P. vivax* to antifolate drugs [23].

The emergence of SP resistance is favored in some patients due to the exposure of the *P. vivax* to the sub-therapeutic levels of SP [37]. The genetic structure of Pvdhfr and Pvdhps was explored. In the Pvdhfr gene, the double mutant haplotype I_13_P_33_F_57_**R**_**58**_T_61_**N**_**117**_I_173_ (S58**R**/ S117**N**) was observed in 22.9% (11/48) and in 20% (17/85) of the isolates in complicated and uncomplicated groups, respectively. In contrast, the majority (78.9%) of the isolates were found to be of wild type (I_13_P_33_F_57_S_58_T_61_S_117_I_173_), with the complete absence of other mutations (F57L, T61S and I173L/F) as reported earlier in the literature [22, 38, 39]. Unlike the previous reports from India, no such triple (**L**57**R**58**N**117) and quadruple (**L**57**R**58**M**61**N**117) mutations were observed [22, 39]. Low nucleotide diversity (π = 0.00182 ± 0.00012) was observed for Pvdhfr as compared to the previous study by Cubides et al. (π = 0.0037) [34]. The overall prevalence of the double mutant haplotypes of Pvdhfr (I_13_P_33_F_57_**R**_**58**_T_61_**N**_**117**_I_173_) obtained was 21% and was comparable to the previous reports from India (38.6 to 40.5%), Pakistan (23–27.4%), Iran (9.5%), and Thailand (35.6%) [35, 40, 41]. A previous study by Hastings et al. has reported the association of a combination of S58**R** and S117**N** with the increased resistance (400 times more than the wild type) to pyrimethamine drug [42]. The results of the predominant presence of the double mutants in the present study was suggestive of the drug pressure on the sympatric *P. vivax* population which might have occurred, due to the use of SP for the treatment of *P. falciparum* infections [27].

Previous literature has also suggested the presence of tandem repeat variant (TRV) polymorphisms in the Pvdhfr gene, which could act as another marker for *P. vivax* SP resistance [43]. On the basis of size polymorphism of the repeat region (TRV), a total of five types were identified. Out of the five types, the predominance of type I (30.8%) consisting of 3 GGDN repeats at amino acid position 88 of Pvdhfr was found. In agreement with previous reports from India, we have also found the exclusive association of the type I (TRV) with the double mutants of Pvdhfr gene in both the groups, whereas the other types were seen to be associated with the wild type alleles [22, 39]. This clearly suggests a more susceptible nature of GGDN repeats getting mutated, thereby developing high levels of resistance. Contrarily Saralamba et al. have reported no association of these allelic types with the point mutations [41]. However, further studies are required to provide a better understanding of the association of TRVs as molecular markers to predict drug resistance and the impact on the infection dynamics [39].

Studies have reported an increased prevalence of mutations in the Pvdhps gene in high SP user areas as compared to low SP user regions. In vitro and in vivo studies have reported the reduced binding affinity of sulphadoxine to Pvdhps containing the double mutation 383**G** 553**G**, as compared to wild type **A**383**A**553 [44]. In the Pvdhps gene, very limited polymorphism was observed, which agrees with the earlier reports from Iran, Lao PDR, India and Colombia [40, 41, 45]. In the present study, only a single SNP (D459A) in Pvdhps was identified among the 15.5 and 5% of the clinical isolates in the complicated and uncomplicated group of patients with the absence of other SNPs (A553G). The presence of D459A was found to be statistically significantly associated (χ2 (1) = 3.904; *p* = 0.04) with the complicated group. The association of D459A SNP with the complications has also been reported earlier by Garg et al. from India where it was found in the patients with hepatic dysfunction. However, out of the novel mutations reported in the group of patients with severe manifestations, F365L and D459A were present far away from the drug binding cavity and had no effect on drug binding as predicted from in silico studies [27]. In view of the previous and present study results on anti-malaria drug resistance surveillance among the complicated and uncomplicated groups, might indicate higher antifolate drug pressure on the *P. vivax* parasites. The analysis of test of neutrality revealed no role of the evolutionary natural selection in the Pvdhfr and Pvdhps drug resistance genes, whereas the statistically significant negative Fu and Li′s D and F test statistic value of Pvmdr-1 gene clearly depicts the selective sweeps (population expansion) might have occurred recently in the *P. vivax* population of the uncomplicated group of patients.

## Conclusion

The observed prevalence of polymorphism is an indicator of beginning trend of the *P. vivax* antimalarial drug resistance. The observed high prevalence of K10 insertion and double mutant haplotype (T958**M** /F1076**L**) among the Pvcrt and Pvmdr-1 gene, makes the situation problematic. Also, SP drug, which is not used directly against *P. vivax*, the observed prevalence of point mutations in the Pvdhfr (S58**R** and S117**N**) and Pvdhps (D459**A**) genes, is relatively high. A significant difference in the prevalence of D459**A** in Pvdhps among the complicated and uncomplicated group of patients suggests drug selection pressure among the complicated group isolates. In the management of severe malaria, malaria control, and elimination programs, the emergence and spread of these multidrug resistance parasites pose a problem. Further regular and synchronized molecular studies are required to identify the significant hubs of drug resistance areas in a country like India where *P. vivax* imposes a major burden, ultimately helping in the management of suitable antimalarial drug policy.

## Supplementary information

**Additional file 1.** Primers for used for the amplification of *Pvcrt-o, Pvmdr-1, Pvdhps and Pvdhfr* genes.

**Additional file 2.** Final concentration of PCR reagents used in nested and conventional PCRs of *Pvcrt-o, Pvmdr-1, Pvdhps* and *Pvdhfr.*

**Additional file 3.** Thermal cycling profile used for the amplification of *Pvcrt-o, Pvmdr-1*, *Pvdhps* and *Pvdhfr genes.*

## Data Availability

The datasets analysed during the current study are available in the present study results.

## Abbreviations

CQ: Chloroquine
MEGA: Molecular Evolutionary Genetics Analysis
DnaSP: DNA Sequence Polymorphism
ACT: Artemisinin combination therapy
SP: Sulphadoxine-pyrimethamine
PGIMER: Postgraduate Institute of Medical Education and Research
crt-o: Chloroquine resistance transporter
DHFR-TS: Dihydrofolate reductase-thymidylate synthase
PPPK-DHPS: Dihydropterin pyrophosphokinase-dihydropteroate synthase
*Pvmdr-1*: *Plasmodium vivax* multidrug resistance gene
Hd: Haplotype diversity
WHO: World Health Organization
SD: Standard deviation
TRV: Tandem repeat variants
