# Supplementary material for: Distribution pattern of amino acid mutations in chloroquine and antifolate drug resistance associated genes in complicated and uncomplicated Plasmodium vivax isolates from Chandigarh, North India
Source: BMC Infect Dis. 2020 Sep 15;20:671. doi: 10.1186/s12879-020-05397-6 (PMC7493319; doi:10.1186/s12879-020-05397-6)
Supplement: Supplementary file 1 — Additional file 1. Primers for used for the amplification of Pvcrt-o, Pvmdr-1, Pvdhps and Pvdhfr genes. [file 12879_2020_5397_MOESM1_ESM.docx]

**Additional File 1:** Primers for used for the amplification of *Pvcrt-o, Pvmdr-1, Pvdhps and Pvdhfr* genes

| **Genes** | **Primers** | **Sequence (5’-3’)** | **Product size (bp)** |
| --- | --- | --- | --- |
| ***Pvcrt-o*** | Outer forward PF | CGCTGTCGAAGAGCC | 1194 |
|  | Outer reverse PR | AGTTTCCCTCTACACCCG |  |
|  | Inner forward NF | TGCATCACGTTATCTGCATC | 1069 |
|  | Inner reverse NR | TCAGCTGCATGACGAAGG |  |
| ***Pvmdr-1*** | Forward | GGA TAG TCA TGC CCC AGG ATT G | 603 |
|  | Reverse | CAT CAA CTT CCC GGC GTA GC |  |
| ***Pvdhps*** | Outer forward PF | ATTCCAGAGTATAAGCACAGCACATTTGAG | 1436 |
|  | Outer reverse PR | CTAAGGTTGATGTATCCTTGTGAGCACATC |  |
|  | Inner forward NF | AATGGCAAGTGATGGGGCGAGCGTGATTGA | 703 |
|  | Inner reverse NR | CAGTCTGCACTCCCCGATGGCCGCGCCACC |  |
| ***Pvdhfr*** | Outer forward PF | ACCGCACCAGTTGATTCCTAC | 1200 |
|  | Outer reverse PR | TGTTAAAGCTGAAGTACACGAG |  |
|  | Inner forward NF | ATGGAGGACCTTTCAGATGT | 785 |
|  | Inner reverse NR | AACGCATTGCAGTTCTCCGA |  |
