# Supplementary material for: Distribution pattern of amino acid mutations in chloroquine and antifolate drug resistance associated genes in complicated and uncomplicated Plasmodium vivax isolates from Chandigarh, North India
Source: BMC Infect Dis. 2020 Sep 15;20:671. doi: 10.1186/s12879-020-05397-6 (PMC7493319; doi:10.1186/s12879-020-05397-6)
Supplement: Supplementary file 2 — Additional file 2. Final concentration of PCR reagents used in nested and conventional PCRs of Pvcrt-o, Pvmdr-1, Pvdhps and Pvdhfr. [file 12879_2020_5397_MOESM2_ESM.docx]

**Additional File 2:** Final concentration of PCR reagents used in nested and conventional PCRs of *Pvcrt-o, Pvmdr-1,* *Pvdhps* and *Pvdhfr*

| **Genes** | **Primer** | |  | | **PCR buffer**  **10X** | | **MgSO_4_50mM** | | **Primer 10µM** | | **dNTP 10mM** | | **Taq polymerase 5U/µL** | | **DNA** | | **Final volume**  **(nuclease free water) µL** | |
| --- | --- | --- | --- | --- | --- | --- | --- | --- | --- | --- | --- | --- | --- | --- | --- | --- | --- | --- |
| ***Pvcrt-o*** | | PF | | Nest1 | | 1X | | 3 | | 0.4 | | 1 | | 0.5 | | 2 | | 12.5 |
|  |  | PR | |  |  |  | |  | |  | |  | |  | |  | |  |
|  |  | NF | | Nest2 | | 1X | | 3 | | 0.4 | | 0.8 | | 0.5 | | 2(1:10) | | 25 |
|  |  | NR | |  |  |  | |  | |  | |  | |  | |  | |  |
| ***Pvmdr-1*** | | F | |  | | 1X | | 3 | | 0.4 | | 0.8 | | 0.5 | | 2 | | 25 |
|  |  | R | |  | |  | |  | |  | |  | |  | |  | |  |
| ***Pvdhps*** | | PF | | Nest1 | | 1X | | 3.5 | | 0.4 | | 1 | | 0.5 | | 2 | | 12.5 |
|  |  | PR | |  |  |  | |  | |  | |  | |  | |  | |  |
|  |  | NF | | Nest2 | | 1X | | 2.5 | | 0.4 | | 0.8 | | 0.5 | | 2(1:10) | | 25 |
|  |  | NR | |  |  |  | |  | |  | |  | |  | |  | |  |
| ***Pvdhfr*** | | PF | | Nest1 | | 1X | | 2.5 | | 0.4 | | 1 | | 0.5 | | 2 | | 12.5 |
|  |  | PR | |  |  |  | |  | |  | |  | |  | |  | |  |
|  |  | NF | | Nest2 | | 1X | | 2.5 | | 0.4 | | 0.8 | | 0.5 | | 2(1:10) | | 25 |
|  |  | NR | |  |  |  | |  | |  | |  | |  | |  | |  |
