# Supplementary material for: Distribution pattern of amino acid mutations in chloroquine and antifolate drug resistance associated genes in complicated and uncomplicated Plasmodium vivax isolates from Chandigarh, North India
Source: BMC Infect Dis. 2020 Sep 15;20:671. doi: 10.1186/s12879-020-05397-6 (PMC7493319; doi:10.1186/s12879-020-05397-6)
Supplement: Supplementary file 3 — Additional file 3. Thermal cycling profile used for the amplification of Pvcrt-o, Pvmdr-1, Pvdhps and Pvdhfr genes. [file 12879_2020_5397_MOESM3_ESM.docx]

**Additional File 3:** Thermal cycling profile used for the amplification of *Pvcrt-o, Pvmdr-1*, *Pvdhps* and *Pvdhfr genes*

| Genes |  |  | 1^st^round (⁰C/minute) | | 2^nd^ round (⁰C/minute) | |
| --- | --- | --- | --- | --- | --- | --- |
| *Pvcrt-o* | Stage 1 | Initial denaturation | 94/7 | | 94/7 | |
|  | Stage 2 | Denaturation | 94/00:30 | 35 rounds | 94/00:30 | 35 rounds |
|  |  | Annealing | 48/00:45 |  | 50.4/00:45 |  |
|  |  | Polymerization | 72/02:00 |  | 72/02:00 |  |
|  | Stage3 | Final extension | 72/7 | | 72/7 | |
| *Pvmdr-1* | Stage 1 | Initial denaturation | 94/5 | |  | |
|  | Stage 2 | Denaturation | 94/00:45 | |  | |
|  |  | Annealing | 62/00:45 39 rounds | |  | |
|  |  | Polymerization | 72/01:30 | |  | |
|  | Stage3 | Final extension | 72/7 | |  | |
| *Pvdhps* | Stage 1 | Initial denaturation | 94/7 | | 94/7 | |
|  | Stage 2 | Denaturation | 94/00:30 | | 94/00:30 | |
|  |  | Annealing | 57/00:45 39 rounds | | 70.6/01:00 35 rounds | |
|  |  | Polymerization | 72/02:00 | | 72/01:30 | |
|  | Stage 3 | Final extension | 72/7 | | 72/7 | |
| *Pvdhfr* | Stage 1 | Initial denaturation | 94/7 | | 94/7 | |
|  | Stage 2 | Denaturation | 94/00:30 | | 94/01:00 | |
|  |  | Annealing | 58/01:00 35 rounds | | 54.4/00:45 39 rounds | |
|  |  | Polymerization | 72/01:00 | | 72/02:00 | |
|  | Stage3 | Final extension | 72/7 | | 72/7 | |
